# Adherence to the World Cancer Research Fund/American Institute for Cancer Research Recommendations and the Risk of Breast Cancer

**DOI:** 10.3390/nu12030607

**Published:** 2020-02-26

**Authors:** Federica Turati, Michela Dalmartello, Francesca Bravi, Diego Serraino, Livia Augustin, Attilio Giacosa, Eva Negri, Fabio Levi, Carlo La Vecchia

**Affiliations:** 1 Unit of Medical Statistics and Biometry, Fondazione IRCCS Istituto Nazionale Dei Tumori Di Milano, via Venezian 1, 20133 Milan, Italy; federica.turati@istitutotumori.mi.it or; 2 Department of Clinical Sciences and Community Health, Università degli studi di Milano, via A. Vanzetti 5, 20133 Milan, Italy; michela.dalmartello@unimi.it (M.D.); francesca.bravi@unimi.it (F.B.); 3 Unit of Cancer Epidemiology, CRO Aviano National Cancer Institute, IRCCS, via F. Gallini 2, 33080 Aviano, Italy; serrainod@cro.it; 4 Unit of Epidemiology, National Cancer Institute, G. Pascale Foundation, via M. Semmola 1, 80131 Naples, Italy; livia.augustin@utoronto.ca; 5 Department of Gastroenterology and Clinical Nutrition, Policlinico di Monza, via Amati 111, 20900 Monza, Italy; attilio.giacosa@gmail.com; 6 Department of Biomedical and Clinical Sciences, Università degli Studi di Milano, via G.B. Grassi 74, 20157 Milan, Italy; eva.negri@unimi.it; 7 Institute of Social and Preventive Medicine (IUMSP), Unisanté, University of Lausanne, Route de la Corniche 10, 1010 Lausanne, Switzerland; fabio.levi@bluewin.ch

**Keywords:** breast cancer, diet, lifestyle, guideline adherence, nutrition, prevention

## Abstract

The World Cancer Research Fund/American Institute for Cancer Research (WCRF/AICR) introduced in 2007, and updated in 2018, nutrition-related recommendations for cancer prevention. Previous studies generally reported inverse associations of breast cancer with the 2007 recommendations, while no study has yet evaluated the association with the 2018 guidelines. We investigated the association between adherence to the 2018 WCRF/AICR recommendations and breast cancer risk in a case–control study from Italy and Switzerland (1991–2008) including 3034 incident histologically-confirmed breast cancer cases and 3392 hospital controls. Adherence to the 2018 guidelines was summarized through a score incorporating eight recommendations (body fatness, physical activity, consumption of wholegrains/vegetables/fruit/beans, “fast foods” and other processed foods high in fat, starches, or sugars, red/processed meat, sugar-sweetened drinks, alcohol, breastfeeding), with higher scores indicating higher adherence. Odds ratios (OR) were estimated using multiple logistic regression models. We also conducted a meta-analysis including 15 additional studies using random-effects models. In our case–control study, adherence to the 2018 WCRF/AICR guidelines was inversely associated with breast cancer, with ORs of 0.60 (95% confidence interval (CI), 0.51–0.70) for a score ≥5.5 vs. ≤4.25, and of 0.83 (95% CI, 0.79–0.88) for a 1-point increment. In our study, 25% of breast cancers were attributable to low-to-moderate guideline adherence. In the meta-analysis, the pooled relative risks (RRs) were 0.73 (95% CI, 0.65–0.82, p heterogeneity among studies < 0.001) for the highest vs. the lowest WCRF/AICR score category, and 0.91 (95% CI, 0.88–0.94, p heterogeneity < 0.001) for a 1-point increment. This work provides quantitative evidence that higher adherence to the WCRF/AICR recommendations reduces the risk of breast cancer, thus opening perspectives for prevention.

## 1. Introduction

After lung, breast cancer is the second most common neoplasm worldwide. In women, it accounts for almost 1 in 4 cancer cases and is the first cause of cancer death in most countries [1,2]. Primary prevention through favorable changes in modifiable risk factors is however a major challenge and a public health priority [3].

Thus far, only very few modifiable risk factors for breast cancer have been recognized, including overweight/obesity, menopausal hormone replacement therapy (HRT), low physical activity and sedentary behavior, with possible differential associations in the life course of women [4,5]. Despite considerable research on the dietary correlates of breast cancer, no consistent evidence exists for specific foods, food groups or nutrients [6], except for the detrimental role of alcohol drinking [7]. Nonetheless, healthy dietary patterns, mainly based on frequent intake of plant-based foods, including the Mediterranean diet [8,9], have been favorably related to breast cancer risk in several studies [10,11,12].

In 2007, the World Cancer Research Fund/American Institute for Cancer Research (WCRF/AICR) published the Second Expert Report, in which defined a set of evidence-based public health recommendations on body fatness, physical activity and diet with the aim of reducing the burden of cancer [13]. In the Third Expert Report, published in 2018, guidelines have been updated according to the latest evidence [14]. While the 2007 and 2018 conclusions and global recommendations look similar, in the most recent report emphasis was placed on the potential benefit of considering recommendations as an integrated pattern of healthy lifestyle behaviors, which taken together have an impact on cancer prevention [15].

Previous studies showed that higher adherence to the 2007 WCRF/AICR nutrition-related recommendations reduced the risk of total cancer and of specific cancers [16]. An inverse association with breast cancer was reported in most—though not all [17,18]—studies, including the European Prospective Investigation into Cancer and Nutrition (EPIC) cohort [19], the Iowa Women’s Health Study (IWHS) [20], the Black Women’s Health Study (BWHS) [21], the Swedish Mammography Cohort (SMC) [22], the Canadian National Breast Screening Study (NBSS) [23], and the Vitamins and Lifestyle (VITAL) cohort [24]. Data are, however, still limited and further quantification is needed. Moreover, no study has yet focused on the most updated version of the WCRF/AICR recommendations.

In the current report, we assessed the relation between adherence to the WCRF/AICR nutrition-related recommendations for cancer prevention and breast cancer risk in a large Mediterranean population, and conducted a meta-analysis of available studies on the topic.

## 2. Materials and Methods

### 2.1. Study Population and Data Collection

Data came from a multicentric case–control study carried out between 1991 and 1994 in 6 Italian areas (i.e., Milan, Genoa, Pordenone/Gorizia, Forlì, Latina, and Naples) and between 1992 and 2008 in the Canton of Vaud, Switzerland [25,26]. Overall, the study included 3034 cases (range: 23–78 years) with incident, histologically confirmed breast cancer, diagnosed within one year before the interview and with no previous cancer, admitted to general hospitals in the study areas. The controls were 3392 women (range: 19–79 years) admitted to the same hospitals as cases for acute, non-neoplastic, non-gynaecological conditions, with no history of cancer and no recent dietary changes. There was no strict age matching, but the age distribution was periodically checked to give a similar age distribution for cases and controls (median age 55 for cases and 56 for controls). Overall, 30% of the controls were admitted for acute surgical conditions, 20% for traumas, 26% for non-traumatic orthopedic conditions, 14% for eye disorders, and 10% for other miscellaneous conditions. The local ethical committees approved the study protocol and study participants gave informed consent, according to the rules in operation at the time of data collection. Less than 5% of the patients approached for interview in Italy and 15% in Switzerland declined participation.

In each centre, a structured questionnaire was administered to both cases and controls by trained interviewers. The questionnaire included questions about sociodemographic factors, dietary and lifestyle habits (e.g., smoking, alcohol intake, and physical activity), anthropometric measurements, menstrual and reproductive factors, a personal medical history, and a family history of cancer. As for anthropometric measures, the questionnaire collected information on body shape at 12 years (thinner, same or heavier than peers of the same age), and self-reported height and average weight at age 30 and 50, as well as before diagnosis (cases) or interview (controls). Body mass index (BMI) was calculated as weight (kg) divided by squared height (m^2^). The interviewers measured the waist circumference (2 cm above the umbilicus). We collected information on occupational/household and leisure time physical activity at different ages (i.e., 12, 15–19, 30–39, and 50–59 years). Cases and controls defined their level of occupational/household physical activity as very heavy, heavy, intermediate, standing or sedentary, depending on their occupation/household activity. Leisure time physical activity was based on the total number of hours of physical activity (e.g., sport, cycling) per week (four different categories: <2 h, 2–4 h, 5–7 h, or >7 h). Women were asked for menopausal status at the time of cancer diagnosis. Information on dietary habits during the two years before diagnosis (for cases) or hospital admission (for controls) was assessed through a food frequency questionnaire (FFQ), which included 78 foods and recipes. The FFQ was tested for its validity and reproducibility with satisfactory results [27,28]. Subjects had to report the average weekly frequency of consumption of each item of the FFQ. Frequencies of 1–3 times per month were coded as 0.5 per week. Daily intakes of selected nutrients and total energy were estimated using an Italian food composition database [29,30].

### 2.2. WCRF/AICR Score

We developed a score measuring the adherence to the 2018 version of the WCRF/AICR guidelines [14], according to the standard scoring approach recently proposed by a collaborative group including, among the others, researchers from the US National Cancer Institute (NCI) and members of AICR and WCRF International [31]. We included 7 general and 1 special WCRF/AICR recommendations on the following aspects: (1) body weight; (2) physical activity; (3) wholegrains, vegetables, fruit, and beans; (4) “fast foods” and other processed foods rich in fats, starches, or sugars; (5) red and processed meat; (6) sugar-sweetened drinks; (7) alcoholic drinks; and (S1) breastfeeding. For each recommendation/sub-recommendation, participants were assigned 1 point for full adherence, 0.5 points for partial adherence, and 0 otherwise, according, whenever possible, to criteria provided by Shams-White et al. [31] (see details in Supplementary Table S1). Information on waist circumference was not available for 173 cases and 381 controls, and was not included in the body weight recommendation. For recommendations with multiple sub-recommendations, we calculated the score as the average of the scores on the sub-recommendations. Therefore, the overall 2018 WCRF/AICR score, calculated by summing the scores of each recommendation, ranged from 0 (minimal adherence) to 8 (maximal adherence). A higher value of the overall score indicated a higher adherence to the guidelines. We also calculated a diet WCRF/AICR score, based on the 5 recommendations on dietary habits; such a diet specific score ranged from 0 to 5. Moreover, we derived a variant of the score without the breastfeeding component, as most previous studies did not include that recommendation. To enable comparison with previous studies, we also constructed a score measuring adherence to the earlier guidelines, as reported in our previous studies [32,33,34].

### 2.3. Statistical Analysis

Odds ratios (OR) of breast cancer and the corresponding 95% confidence intervals (CI) for each individual recommendation, for the overall WCRF/AICR score, and for the diet WCRF/AICR score were estimated using unconditional multiple logistic regression models. We fitted a first model with terms for age, study centre, and education, and a second one with further terms for parity, menopausal status and age at menopause, use of oral contraceptive and HRT, tobacco smoking, non-alcoholic energy intake (according to quintiles among controls), family history of breast cancer, and diabetes. When evaluating the association with individual recommendations, analyses were also adjusted for BMI, physical activity, and alcohol intake (unless the covariate was the recommendation under evaluation). We performed tests for trend including the variable in its ordinal form. In addition, we carried out a sensitivity analysis by excluding alternately each component in turn from the original 2018 WCRF/AICR score to evaluate the relative importance of each recommendation. The potential effect modification of menopausal status was tested using a likelihood ratio test comparing the likelihoods of models with and without terms for interactions between menopausal status and the WCRF/AICR score variable. We also estimated the population attributable fraction, representing the proportion of breast cancers that would be avoided if the whole population were highly adherent to the WCRF/AICR recommendations [35].

The aforementioned analyses were conducted using the SAS software, version 9.4 (SAS Institute, Inc., Cary, NC, USA).

### 2.4. Meta-Analysis

Using PubMed with terms breast, cancer/neoplasm/carcinoma and World Cancer Research Fund/WCRF in October 2019, we identified observational studies on the association between adherence to the WCRF/AICR guidelines (measured by means of an a priori score) and the risk of or mortality from breast cancer. Fifteen publications were identified and considered in the meta-analysis [8,17,18,19,20,21,22,23,24,36,37,38,39,40,41]. These included a pooled analysis [39] focusing on dietary recommendations only and combining data from the elderly segments of 5 EPIC cohorts, the NIH-AARP study, and the Rotterdam study. To avoid overlap with the overall EPIC results provided in Romaguera et al. [19], we extracted from that pooled-analysis only study-specific relative risks (RRs) from the NIH-AARP cohort and from the Rotterdam study. One study on in situ breast cancer was excluded [42]. All studies investigated adherence to the 2007 WCRF/AICR recommendations. We extracted from original studies RR estimates with the higher degree of adjustment. In the study by Nomura et al. [21] we extracted results for the baseline score rather than those from the time-varying score, for consistency with other studies.

We calculated the pooled RR and the corresponding 95% CI by means of fixed or random effects models, based on the results of the Cochrane Q test (heterogeneity was defined as *p* < 0.1) [43]. Inconsistency among studies was quantified using the I^2^ statistics [44] that measures the percentage of variability that cannot be attributed to random error (I^2^ > 50% representing substantial heterogeneity). We conducted both an extreme quantile meta-analysis (i.e., comparing the highest vs. lowest category of the WCRF/AICR score) and a linear dose-response meta-analysis (i.e., per 1-point increase in the score). Studies were included in the extreme quantile and/or dose-response meta-analyses based on data availability. One study providing only the RR for 1-SD increase in the score was also considered in the dose-response meta-analysis [8]. For the dose-response meta-analysis, we derived the estimate of the RR for 1-point increment in the score when studies provided results for a different increment [20,21,37] or from the categorical analysis only [41]. In the latter scenario, the weighted least squares regression method was used, as the number of cases and non-cases across score categories was not available in the original publication [41]. From the study by Castello et al. [37] we derived the estimate of the RR for the highest vs. the lowest score category by changing the reference category. The publication bias was evaluated by visual inspection of the funnel plot and by the Begg test [45]. A sensitivity analysis by excluding each study one by one was also conducted.

The statistical analyses for meta-analysis were conducted using the STATA software (version 14; StataCorp, College Station, TX, USA).

## 3. Results

The distribution of selected characteristics in breast cancer cases and controls is shown in Table 1. Compared to controls, cases were more educated, had lower parity and had more frequently a first-degree relative with breast cancer.

Adherence to the recommendations on physical activity, wholegrains, vegetables, fruit and beans, fast foods and other processed food high in fat starches or sugar, sugar sweetened drinks, and alcohol was associated with a significant lower risk of breast cancer, with significant decreasing trends for increasing adherence (Table 2). Adherence to the recommendation on red and processed meat was significantly inversely associated with breast cancer in the analyses adjusted for age, centre, and education, while results were marginally significant in fully adjusted analyses. No association was found for adherence to the recommendations on body weight and breastfeeding.

Table 3 provides the ORs of breast cancer according to the overall 2018 WCRF/AICR score and the diet-specific score. There was an inverse association with the WCRF/AICR adherence score, with an OR of 0.60 (95% CI: 0.51–0.70) for a score ≥5.5 vs. ≤4.25 in the fully adjusted analysis (p for trend < 0.001). The results were consistent according to menopausal status (Supplementary Table S2). The inverse association was observed in both countries, but was apparently stronger in Switzerland. The ORs for successive score categories were 0.81 (95% CI: 0.69–0.95), 0.83 (95% CI: 0.72–0.97) and 0.74 (95% CI: 0.62–0.88) in Italy, and 0.55 (95% CI: 0.38–0.80), 0.61 (95% CI: 0.44–0.86) and 0.23 (95% CI: 0.15–0.34) in Switzerland. A 1-point increment in the score decreased breast cancer risk by 17% (OR = 0.83, 95% CI: 0.79–0.88). According to the estimated population attributable fraction, 25% of breast cancers could be avoided in our population if all participants would shift towards the highest adherence category. The score based on dietary recommendations only was significantly inversely related to breast cancer risk, too (OR = 0.62, 95% CI: 0.53–0.73 for a score >3.5 vs. <2.25, p for trend < 0.001).

Similar results (but apparently less strong) were observed when analyzing the 2007 version of the score (Supplementary Table S3). The ORs were 0.67 (95% CI, 0.56–0.79) for the highest vs. the lowest score category (p for trend < 0.001) and 0.85 (95% CI, 0.80–0.90) for a 1-point increment in the score.

In a sensitivity analysis, consistent results were found when omitting each recommendation one by one from the score: the ORs comparing extreme categories varied between 0.54 and 0.69. In particular, the OR was 0.56 (95% CI, 0.44–0.72) when removing from the score the recommendation on breastfeeding.

### Meta-Analysis

**Figure 1:**
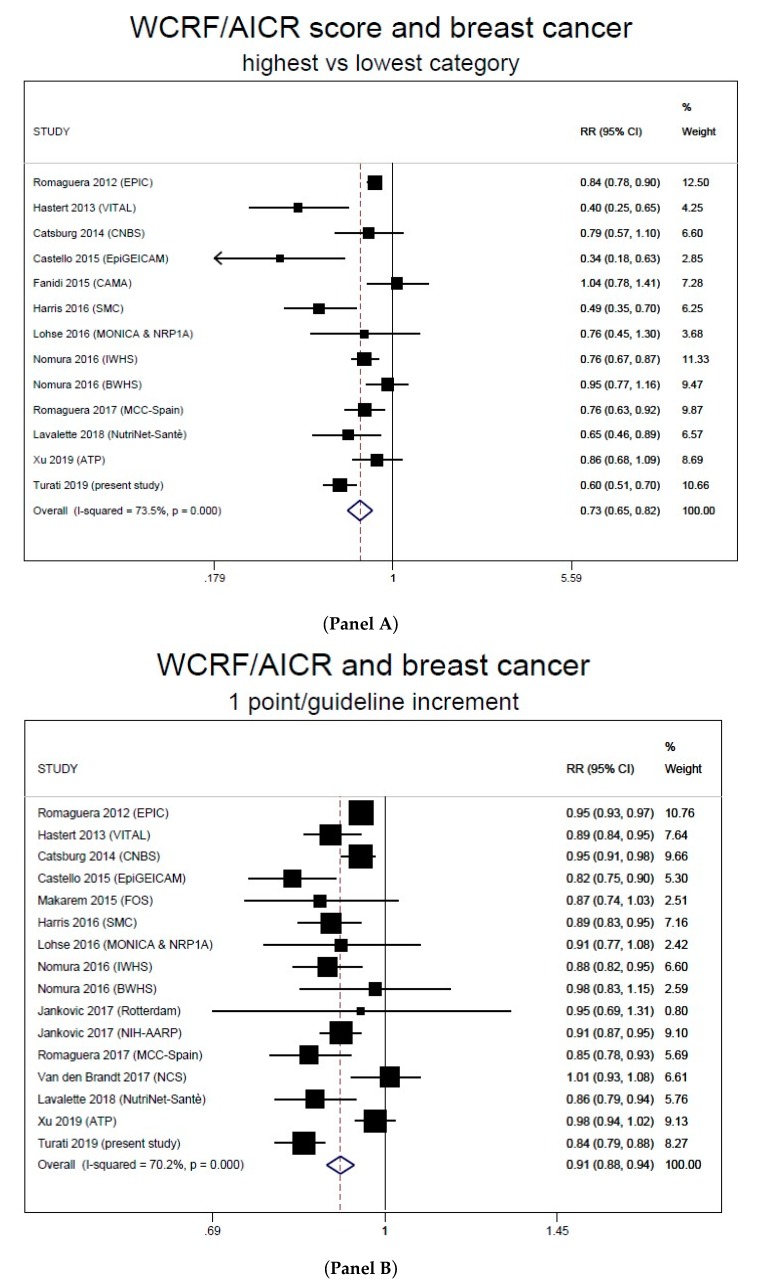
Forest plots for the extreme-quantile (**Panel A**) and linear dose-response (**Panel B**) meta-analyses on breast cancer and the WCRF/AICR score. WCRF/AICR, World Cancer Research Fund/American Institute for Cancer Research.

The description of the studies included in the meta-analysis is provided in Table 4. Based on 13 studies, including the present one, the pooled RR of breast cancer for the highest vs. the lowest category of the WCRF/AICR score was 0.73 (95% CI, 0.65–0.82, I^2^ = 73.5%) (Figure 1 panel A). Summary estimates were similar for case–control (RR 0.68, 95% CI, 0.51–0.92, p heterogeneity among studies = 0.001, I^2^ = 81.7%, 4 studies) and cohort studies (RR 0.75, 95% CI, 0.66–0.85, p heterogeneity = 0.004, I^2^ = 64.8%, 9 studies), and for pre- (RR 0.70, 95% CI, 0.47–1.03, 5 studies) and post-menopausal women (RR 0.67, 95% CI, 0.56–0.80, 8 studies). When we included for the study by Nomura et al. [21] the results based on the time-varying score rather than those based on the baseline score, the pooled RR became 0.72 (95% CI, 0.64–0.81). In the sensitivity analysis, no single study appreciably influenced the summary results, with the pooled RR comparing the extreme score categories ranging from 0.71 to 0.75 (significant) when omitting individual studies one at a time.

In the linear dose-response meta-analysis, each 1-point increase in the score was associated to a 9% (95% CI, 6%–12%, I^2^ = 70.2%) RR reduction, based on 16 studies (15 publications) including the present one (Figure 1, panel B). The pooled RRs were 0.93 (95% CI, 0.91–0.95, p heterogeneity = 0.022, I^2^ = 49.4%) for cohort and 0.83 (95% CI, 0.80–0.87, p heterogeneity = 0.847, I^2^ = 0%) for case–control studies. The exclusion of each study in turn did not appreciably influence the summary result, and the estimated RRs were always significant. In particular, the pooled RR was still 0.91 (95% CI, 0.88–0.94) when omitting the pooled analysis by Jancovic et al. [39], which considered only dietary recommendations, and 0.90 (95% CI, 0.87–0.93) when omitting the study by Van de Brandt [8], which reported the RR for 1-SD increase in a dietary WCRF/AICR score.

Since all previous studies assessed adherence to the 2007 WCRF/AICR recommendations, as sensitivity analysis, we replaced in the meta-analyses our results for the 2018 score with those for the 2007 score. We obtained very similar summary estimates (RR for the extreme quantile meta-analysis: 0.74, 95% CI, 0.66–0.83, RR for the linear dose-response meta-analysis: 0.91, 95% CI, 0.89–0.94).

No significant publication bias was detected (*p* = 0.143).

## 4. Discussion

In the present large case–control study, higher adherence to the diet, adiposity, and physical activity recommendations provided by the WCRF/AICR Third Expert Report was associated with reduced breast cancer risk, independently of menopausal status. The results from the sensitivity analysis suggest that the association was not driven by adherence to one specific recommendation, but rather by the combined and synergic effects of the various score components. The meta-analysis indicates that women who highly adhere to the recommendations had an approximately 30% lower risk of breast cancer compared to those with low adherence and that each 1-point increment in the WCRF/AIRC score is associated with a significant 9% reduced risk. 

Published studies vary in design, baseline population characteristics, data collection, number and definition of recommendations operationalized in the score, and adjustment factors; the observed heterogeneity is therefore not surprising. In any case, they are largely supportive of the preventing role of following the WCRF/AICR guidelines on breast cancer. Indeed, estimates of the RR of breast cancer comparing extreme score categories were below unity in all but one studies [18], and were significant in seven out of the 12 previous studies. The favorable role of adherence to the recommendations was evident even when investigating the association continuously, with significantly reduced RRs in nine out of 15 previous studies. RRs were below unity in all the remaining five studies except for one [8].

Only a few reports investigated the association between the 2018 release of the WCRF/AICR recommendations and cancer risk [42,46,47,48]. The present study is the first evaluating the updated recommendations in the prevention of invasive breast cancer. The 2007 and 2018 sets of recommendations were similar, and consequently major differences in terms of associated cancer risk reductions are unlikely. The last release of the guidelines recommends the avoidance of any alcohol (unlike the previous version, which allowed moderate consumption), included avoidance of sugar-sweetened drinks as a separate recommendation, and removed the 2007 recommendation on limiting salt intake and avoid moldy cereals or pulses from the global set of recommendations (moving it into a regional and special circumstances section, based on the fact that salt-preserved food is mostly consumed by people without access to refrigeration and evidence on salt-preserved foods consumption as a risk factor for cancer derived mainly from selected Asian populations). Alcohol has been directly associated to breast cancer even at low amounts [49]. According to the 2018 alcohol recommendation, we observed a breast cancer risk reduction of 26% among non-drinkers (adherent) vs. women drinking over seven drinks per week. When evaluating the earlier recommendation, the protection associated to adherence (defined as ≤7 drinks/week) was slightly lower, i.e., 21% (OR: 0.79, 95% CI, 0.68–0.92 vs. >14 drinks/week). Thus, adherence to the stricter updated recommendation has the potential to further reduce the incidence of breast cancer. Conversely, since salt-preserved food is uncommon among Western populations and its consumption has been unfavorably related to stomach cancer only, the change in the corresponding recommendation is likely to have a negligible impact on breast cancer risk reduction. The association of sugar-sweetened drink consumption with breast cancer is limited [50,51] (although further quantification is needed). It is difficult to assess the added benefit of the updated recommendation on breast cancer in our database, since sugar-sweetened drinks consumption was relatively uncommon in our population at the time of data collection. We found similar results when using scores measuring overall adherence to the 2007 and 2018 guidelines, and our findings are largely in agreement with previous studies relying on recommendations from the earlier WCRF/AICR report. Along this line, recent findings on the association between the 2018 WCRF/AICR recommendation and colorectal cancer risk from the Nurses’ Health Study and Health Professionals Follow-up Study confirmed the results of prior studies based on the 2007 guidelines release [47].

Our findings are in agreement with the conclusions of a systematic review [16] and with studies assessing compliance to the American Cancer Society (ACS) Nutrition and Physical Activity Cancer Prevention Guidelines (which largely overlap with those from the WCRF/AICR), which reported lower risks of breast cancer [23,52,53] and other selected cancers [52,53] for higher scores of guideline adherence. Recently, data from the EPIC cohort showed a favorable role of adherence to the 2018 WCRF/AICR recommendations on in situ breast cancer [42]—a precursor of invasive breast cancer, with which it shares some of the risk factors [54]—in the subgroup of women enrolled through screening programs and with high screening participation during follow-up.

In our case–control study, we observed a significant inverse association between a score reflecting adherence to the WCRF/AIRC dietary recommendations and breast cancer, in agreement with the study by Lavalette et al. [36], which reported an hazard ratio (HR) of 0.83 (95% CI, 0.74–0.93) for 1-point increment in a WCRF/AICR score without the body fatness and physical activity components (as well as the component relying on breastfeeding). Other studies found non-significant or borderline significant inverse associations [21,39] or null associations with dietary WCRF/AICR scores [8,20]. Despite the large number of investigations, evidence on specific aspects of diet (single foods/nutrients, food groups, and dietary components) and breast cancer is still open to discussion, with the exception of the widely recognized detrimental role of alcohol [6]. According to the Continuous Update Project (CUP) by the WCRF/AICR, only limited evidence exists that the consumption of non-starchy vegetables, dairy products, and foods containing carotenoids and calcium decreases the risk of pre-menopausal and/or post-menopausal breast cancer [55]. Meanwhile, studies investigating healthy dietary patterns, such as the Mediterranean diet [8,9], have generally showed inverse associations [10], pointing to the importance of the overall diet quality as compared to individual foods/food components in breast cancer prevention. Dietary patterns capture the complexity of the diet, account for the interactions among dietary factors, and are more predictive of disease risk when single foods/food components have modest health effects [56].

To improve the comparability of our results with those from future studies relying on the 2018 WCRF/AICR guidelines, in the construction of the score, whenever possible, we followed the standard scoring approach recently proposed by a NCI-led collaborative group, which included, among the others, researchers from AICR and WCRF International [31]. However, (1) we did not include information on waist circumference in the assessment of the recommendation on body weight, as data were incomplete (not available for 173 cases and 381 controls); (2) we adapted the definition of no, partial, and full adherence to the recommendation on physical activity according to the information collected from our questionnaire; (3) we used energy-density as a proxy of the % of total energy intake from ultra-processed foods (we did not collect specific information on ultra-processed foods, as their consumption was relatively infrequent in our population at the time of data collection); and (4) we could not distinguish between exclusive and partial breastfeeding. As for point (1), when we used data on both waist circumference and BMI in the sample of subjects with information on both factors we obtained very similar results (OR for full vs. no adherence to the recommendation on body weight = 1.03, 95% CI, 0.89–1.20, OR for the highest vs. the lowest WCRF/AICR score = 0.56, 95% CI, 0.48–0.66).

The current analysis is based on data collected between 1991 and 2008 while looking at associations with more recent recommendations. Thus, the observation that the protection associated to the adherence to the WCRF/AICR recommendations was found in a population unaware of those guidelines supports the absence of major information bias and hence the validity of the association. The data were collected at multiple centres across two countries. However, similar structured questionnaires and coding manuals were used in all study centres, and interviewers were centrally trained, thus improving the reliability of data collection across study centers. In addition, a comparison across countries indicated a similar distribution of age, menopausal status, and family history of breast cancer, while Swiss women were more educated and more often nulliparae than Italian ones [9]. A favorable role on breast cancer of adherence to the guidelines was observed in Italian and Swiss centers, although apparently stronger in the latter’s, supporting the consistency of the observed association.

Hospital controls may have different dietary habits from those of the general population, but we minimized bias by excluding from the control series patients admitted to hospital for conditions which may have led to long-term diet modifications. The strengths of the present case–control study lie in the satisfactory reproducibility and validity of the FFQ [27,28,57], the similar catchment area of cases and controls, the almost complete participation rate, and the large sample size. However, measuring usual diet is challenging and some degree of exposure misclassification cannot be excluded. Since weight and height were self-reported, BMIs are likely underestimated. However, information bias is likely to be similar in cases and controls. In addition, although we adjusted our risk estimates for several exposures, including hormone-related factors, diabetes, family history of breast cancer and total energy intake, some residual confounding is still possible.

In conclusion, the present work provides quantitative evidence that greater adherence to the nutrition-related WCRF/AICR recommendations is favorably related to breast cancer risk, and thus suggests that encouraging adherence to the guidelines is a valuable public health strategy for breast cancer prevention.

## Figures and Tables

**Table 1:** Distribution of 3034 cases of breast cancer and 3392 controls according to selected characteristics. Italy and Switzerland, 1991–2008.

Characteristic	Cases	Controls
	*n* (%)	*n* (%)
Centre		
Pordenone/Gorizia	1046 (34.5)	1015 (29.9)
Milan	585 (19.3)	623 (18.4)
Genoa	290 (9.6)	310 (9.1)
Forlì	212 (7.0)	213 (6.3)
Naples	258 (8.5)	249 (7.3)
Rome/Latina	178 (5.9)	178 (5.3)
Switzerland	465 (15.3)	804 (23.7)
Age group		
<45	562 (18.5)	686 (20.2)
45–54	898 (29.6)	870 (25.7)
55–64	912 (30.1)	978 (28.8)
≥65	662 (21.8)	858 (25.3)
Menopausal status ^a^		
Premenopause	1150 (38.0)	1180 (34.8)
Postmenopause	1880 (62.1)	2212 (65.2)
Education (years) ^a^		
<7	1273 (42.2)	1583 (47.0)
7–11	972 (32.2)	1120 (33.2)
≥12	775 (25.7)	666 (19.8)
Parity ^a^		
Nulliparae	504 (16.6)	597 (17.6)
1	676 (22.3)	688 (20.3)
2	1163 (38.4)	1179 (34.8)
≥3	688 (22.7)	926 (27.3)
Family history of breast cancer in first-degree relatives		
No	2724 (89.8)	3249 (95.8)
Yes	310 (10.2)	143 (4.2)

^a^ The sum does not add up to the total because of missing values.

**Table 2:** Odds ratios (ORs) and corresponding 95% confidence intervals (CIs) of breast cancer for individual 2018 WCRF/AICR recommendations. Italy and Switzerland, 1991–2008.

	Cases (%)	Controls (%)	OR (95% CI) ^a^	OR (95% CI) ^b^
R1—Be a healthy weight ^c^				
0	505 (16.7)	589 (17.4)	1.00 ^d^	1.00^d^
0.5	960 (31.7)	1128 (33.3)	0.99 (0.85–1.15)	0.97 (0.83–1.13)
0.5	1562 (51.6)	1667 (49.3)	1.01 (0.88–1.17)	0.99 (0.86–1.15)
*p_trend_*			*0.763*	*0.984*
R2—Be physically active ^c^				
0	223 (7.4)	149 (4.4)	1.00 ^d^	1.00 ^d^
0.5	2098 (69.4)	2207 (65.5)	0.75 (0.60–0.94)	0.76 (0.60–0.95)
1	702 (23.2)	1016 (30.1)	0.60 (0.47–0.76)	0.61 (0.48–0.78)
*p_trend_*			*<0.001*	*<0.001*
R3—Eat a diet rich in wholegrains vegetables fruit and beans				
<0.5	356 (11.7)	452 (13.3)	1.00 ^d^	1.00 ^d^
0.5–<1	2176 (71.7)	2421 (71.4)	1.03 (0.88–1.20)	0.79 (0.66–0.94)
1	502 (16.5)	519 (15.3)	1.03 (0.85–1.25)	0.63 (0.50–0.80)
*p_trend_*			*0.793*	*<0.001*
R4—Limit consumption of fast foods and other processed food high in fat starches or sugar				
0	456 (15.0)	419 (12.4)	1.00 ^d^	1.00 ^d^
0.5	1752 (57.7)	1807 (53.3)	0.90 (0.78–1.05)	0.95 (0.81–1.11)
1	826 (27.2)	1166 (34.4)	0.67 (0.57–0.79)	0.75 (0.63–0.90)
*p_trend_*			*<0.001*	*<0.001*
R5—Limit consumption of red meat and processed meat				
0	2236 (73.7)	2451 (72.3)	1.00 ^d^	1.00 ^d^
0.5	655 (21.6)	703 (20.7)	0.91 (0.80–1.03)	1.04 (0.91–1.19)
1	143 (4.7)	238 (7.0)	0.66 (0.53–0.82)	0.81 (0.64–1.02)
*p_trend_*			*<0.001*	*0.329*
R6—Limit consumption of sugar sweetened drinks ^c^				
0	187 (6.2)	148 (4.4)	1.00 ^d^	1.00 ^d^
0.5	1147 (37.8)	1216 (35.8)	0.72 (0.57–0.91)	0.74 (0.58–0.94)
1	1700 (56.0)	2028 (59.8)	0.61 (0.48–0.76)	0.68 (0.53–0.86)
*p_trend_*			*<0.001*	*0.003*
R7—Limit alcohol consumption				
0	1081 (35.7)	989 (29.2)	1.00 ^d^	1.00 ^d^
0.5	937 (30.9)	937 (27.7)	0.92 (0.81–1.05)	0.91 (0.80–1.04)
1	1014 (33.4)	1462 (43.2)	0.70 (0.62–0.79)	0.74 (0.65–0.84)
*p_trend_*			*<0.001*	*<0.001*
S1—For mothers, breastfeed if you can ^c^				
0	1083 (35.8)	1194 (35.3)	1.00 ^d^	1.00 ^d^
0.5	602 (19.9)	571 (16.9)	1.09 (0.95–1.26)	1.02 (0.86–1.20)
0.5	1342 (44.3)	1613 (47.8)	0.97 (0.86–1.08)	0.96 (0.83–1.11)
*p_trend_*			*0.512*	*0.496*

WCRF/AICR, World Cancer Research Fund/American Institute for Cancer Research. ^a^ Adjusted for age, study centre, and education. ^b^ Adjusted for age, study centre, education, parity, menopausal status and age at menopause, oral contraceptive use, hormone replacement therapy use, tobacco smoking, non–alcoholic energy intake, family history of breast cancer, diabetes, and additionally for body mass index, physical activity, and alcohol intake unless the variable was part of the recommendation under evaluation. ^c^ The sum does not add up to the total because of missing values. ^d^ Reference category.

**Table 3:** Odds ratios (ORs) and corresponding 95% confidence intervals (CIs) for breast cancer according to the overall 2018 WCRF/AICR score and the diet-specific WCRF/AICR score. Italy and Switzerland, 1991–2008.

	Cases (%)	Controls (%)	OR (95% CI) ^a^	OR (95% CI) ^b^
Overall WCRF/AICR score ^c^				
≤4.25	1103 (36.7)	939 (28.1)	1.00 ^d^	1.00 ^d^
>4.25–<4.75	577 (19.2)	676 (20.2)	0.74 (0.64–0.85)	0.75 (0.65–0.87)
4.75–<5.5	838 (27.9)	964 (28.8)	0.76 (0.67–0.87)	0.79 (0.69–0.90)
≥5.5	490 (16.3)	768 (22.9)	0.57 (0.49–0.66)	0.60 (0.51–0.70)
*p_trend_*			*<0.001*	*<0.001*
WCRF, a increment unit			0.82 (0.78–0.86)	0.83 (0.79–0.88)
Diet WCRF/AICR score ^c^				
<2.25	744 (24.5)	636 (18.8)	1.00 ^d^	1.00 ^d^
2.25–3	1183 (39.0)	1231 (36.3)	0.82 (0.72–0.94)	0.84 (0.73–0.96)
3–3.5	603 (19.9)	787 (23.2)	0.66 (0.57–0.77)	0.71 (0.60–0.83)
>3.5	502 (16.6)	734 (21.7)	0.58 (0.49–0.68)	0.62 (0.53–0.73)
*p_trend_*			*<0.001*	*<0.001*

WCRF/AICR, World Cancer Research Fund/American Institute for Cancer Research. ^a^ Adjusted for age, study centre, and education. ^b^ Adjusted for age, study centre, education, parity, menopausal status and age at menopause, oral contraceptive use, hormone replacement therapy use, smoking, non-alcoholic energy intake, family history of breast cancer, and diabetes. ^c^ The sum does not add up to the total because of missing values. ^d^ Reference category.

**Table 4:** Characteristics of the studies included in the meta-analysis.

Author	Region	Study Design	Study Period	BC Cases/Person at Risk or Controls	Recommendations ^ǁ^ in the WCRF/AICR Score									Extreme Categories Compared
					1	2	3	4	5	6	7	8	9	
Romaguera 2012 [19]	Europe	Cohort EPIC	from 1992 mean f-u: 11 yrs	9358/386,355	x	x	x	x	x	x			x	6–7 vs. 0–3
Hastert 2013 [24]	USA	Cohort VITAL	2000–2008	899/30,797 postmenopausal	x	x	x	x	x	x				5–6 vs. 0
Catsburg 2014 [23]	Canada	Cohort NBSS	1982–2000	2503/49,613	x	x	x	x	x	x	x			6–7 vs. 0–1
Castello 2015 [37]	Spain	Case–control EpiGEICAM	2006–2011	973/973	x	x	x	x	x	x	x	x		0–<3 vs. 6–9 ^§^
Fanidi 2015 [18]	Mexico	Case–control CAMA	2004–2007	1000/1074	x	x	x	x	x	x			x	IV vs. I (<3.25) quartile
Makarem 2015 [38]	USA	Cohort FOS	1991–2008	124/2983	x	x	x	x	x	x	x			NA ^¥^
Harris 2016 [22]	Sweden	Cohort SMC	1997–2012	1388/31,514 mostly postmenopausal	x	x	x	x	x	x	x	x		6–7 vs. 0–2
Lohse 2016 [17]	Switzerland	Cohort MONICA & NRP1A	MONICA: from 1983 NRP1A: from 1977 mean f-u: 21.7 yrs	1332 */16,722 * deaths from BC	x	x	x	x	x	x	x			5–9 vs. 0–3.5
Nomura 2016 [20]	USA	Cohort IWHS	1986–2010	3189/36,626 postmenopausal	x	x	x	x	x	x	x			6–8 vs. 0–3.5
Nomura 2016 [21]	USA	Cohort BWHS	1995–2011	1827/49,103	x	x	x	x	x	x	x			4–7 vs. 0–3
Jancovic 2017 [39]	Worldwide	Pooled analysis of cohort studies EPIC-Elderly NIH-AARP Rotterdam Study	1988–2011	6994/362,114 >60 yrs postmenopausal			x	x	x	x				NA ^¥^
Romaguera 2017 [40]	Spain	Case–control MCC-Spain	2007–2012	1343/3431	x	x	x	x	x	x				III vs. I tertile
Van den Brandt 2017 [8]	The Netherlands	Cohort NLCS	1986–2007	2321/1665 ^ postmenopausal			x	x	x	x	x			NA ^¥^
Lavalette 2018 [36]	France	Cohort NutriNet-Santè	2009–2017	488/41,547	x	x	x	x	x	x	x	x		V vs. I quintile
Xu 2019 [41]	Canada	Cohort ATP	2001–	454/15,787	x	x		x	x	x		x		4–6 vs. 0–2

^ case-cohort analyses based on 2321 BC cases and 1665 subcohort members randomly sampled from the baseline cohort of 62,573 women. ^§^ The odds ratio (OR) provided in the original publication was for 0–<3 vs. 6–9 points (reference category). We calculated the OR for 6–9 vs. 0–<3 points in the score to include the study in the extreme quantile meta-analysis. ^¥^ Only results from the continuous analysis were available. **^ǁ^** Recommendations according to the 2007 WCRF/AICR report: 1: Body fatness, 2: Physical activity, 3: Foods and drinks that promote weight gain, 4: Plant foods, 5: Animal foods, 6: Alcohol, 7: Preservation, processing, preparation, 8: Supplements, 9: Breastfeeding (special recommendation).

## Supplementary Materials

The following are available online at https://www.mdpi.com/2072-6643/12/3/607/s1, Table S1: Operationalization of the 2018 WCRF/AICR recommendations, Table S2: Odds ratios (ORs) and corresponding 95% confidence intervals (CIs) for breast cancer according to the overall 2018 WCRF/AICR score across strata of menopausal status, Table S3: Odds ratios (ORs) and corresponding 95% confidence intervals (CIs) for breast cancer according to the overall 2007 WCRF/AICR score.
