# Supplementary material for: Adherence to the World Cancer Research Fund/American Institute for Cancer Research Recommendations and the Risk of Breast Cancer
Source: Nutrients. 2020 Feb 26;12(3):607. doi: 10.3390/nu12030607 (PMC7146587; doi:10.3390/nu12030607)
Supplement: Supplementary file 1 [file nutrients-12-00607-s001.pdf]

Supplementary Table S1. Operationalization of the 2018 WCRF/AICR recommendations.

2018 WCRF/AICR recommendations	2018 WCRF/AICR Score [1]	Points	Operationalization/Comments	Points
1) Be a healthy weight.	**BMI (kg/m²):**		**BMI (kg/m²):**	
	18.5 – 24.9	0.5	18.5 – 24.9	1
	25 – 29.9	0.25	25 – 29.9	0.5
	<18.5 or ≥30	0	<18.5 or ≥30	0
	**Waist circumference (cm):**		**Waist circumference (cm):**	
	Men: <94	0.5	Insufficient data[a]	-
	Women: <80			
	Men: 94-<102	0.25		
	Women:80-<88			
	Men: ≥102	0		
	Women: ≥88			
2) Be physically active.	**Total moderate-vigorous physical activity (min/week):**		**Total moderate-vigorous physical activity:**	
	≥150	1	Very Heavy/Heavy job[b] or ≥5 hours/week of leisure time physical activity (at age 30-39 years)	1
	75-<150	0.5	Medium job[b] and ≤4 hours/week of leisure time physical activity, or Standing/Sedentary job and 2-4 hours/week of leisure time physical activity (at age 30-39 years)	0.5
	<75	0	Sedentary job[b] and <2 hours/week of leisure time physical activity (at age 30-39 years)	0
3) Eat a diet rich in wholegrains, vegetables, fruit and beans[c].	**Fruits and vegetables (g/day):**		**Fruits and vegetables (g/day):**	
	≥400	0.5	≥400	1
	200-<400	0.25	200-<400	0.5
	<200	0	<200	0
	**Total fiber (g/day):**		**Total fiber (g/day):**	
	≥30	0.5	≥30	1
	15-<30	0.25	15-<30	0.5
	<15	0	<15	0
4) Limit consumption of “fast foods” and other processed foods high in fat, starches or sugars	**Percent of total kcal from ultra-processed foods:**		**Energy dense foods (g/day):**	
	Tertile 1	1	Energy density: ≤125 kcal/100 g/day	1
	Tertile 2	0.5	Energy density: >125 to <175 kcal/100 g/day	0.5
	Tertile 3	0	Energy density: ≥175 kcal/100 g/day	0
5) Limit consumption of red and processed meat.	**Total red meat (g/week) and processed meat (g/week):**		**Red and processed meat (g/wk) and processed meat (g/week):**	
	Red meat <500 and processed meat <21	1	Red and processed meat <500 and processed meat <21	1
	Red meat <500 and processed meat 21-<100	0.5	Red and processed meat <500 and processed meat 21-<100	0.5

	Red meat >500 or processed meat ≥100	0	Red and processed meat ≥500 or processed meat ≥100	0
6) Limit consumption of sugar sweetened drinks	**Total sugar-sweetened drinks (g/day)**		**Total sugar-sweetened drinks (g/day)**	
	0		0	1
	0-≤250		0-≤250	0.5
	>250		>250	0
7) Limit alcohol consumption	**Total ethanol (g/day):**		**Alcoholic drinks (n/week):**	
	0	1	0	1
	>0 and ≤28 (2 drinks) for males and >0 and ≤14 (1 drink) for females	0.5	>0 and ≤14 for males and >0 and ≤7 for females	0.5
	>28 (2 drinks) for males and >14 (1 drink) for females	0	>14 for males and >7 for females	0
8) (Optional) For mothers: breastfeed your baby, if you can	**Exclusive breastfeeding over lifetime for a total of:**		**Cumulative breastfeeding for a total of:**	
	≥6 months	1	≥6 months	1
	>0 - <6 months	0.5	>0 - <6 months	0.5
	Never	0	Never	0

[a] Data on waist circumference were not available for 173 cases and 381 controls. [b] Job and/or household activity. [c] The scoring of the recommendation was calculated as the mean of the sub-recommendation. AICR: American Institute for Cancer Research; BMI: body mass index; WCRF: World Cancer Research Fund.

[1] Shams-White, M.M.; Brockton, N.T.; Mitrou, P.; Romaguera, D.; Brown, S.; Bender, A.; Kahle, L.L.; Reedy, J. Operationalizing the 2018 world cancer research fund/american institute for cancer research (wcrf/aicr) cancer prevention recommendations: A standardized scoring system. *Nutrients* **2019**, *11*.

Supplementary Table S2. Odds ratios (ORs) and corresponding 95% confidence intervals (CIs) for breast cancer according to the overall 2018 WCRF/AICR score across strata of menopausal status. Italy and Switzerland, 1991-2008.

	**Premenopausal**		Postmenopausal	
	**cases/ controls**	OR (95% CI) [a]	cases/ controls	OR (95% CI) [a]
Overall 2018 WCRF/AICR score [c]				
≤ 4.25	477/361	1.00 [b]	624/578	1.00 [b]
>4.25-<4.75	224/235	0.76 (0.60-0.97)	352/441	0.76 (0.63-0.92)
4.75-<5.5	301/320	0.76 (0.60-0.95)	537/644	0.81 (0.68-0.96)
≥ 5.5	138/244	0.48 (0.36-0.63)	352/524	0.68 (0.56-0.82)
p heterogeneity=0.339				

WCRF/AICR, World Cancer Research Fund/American Institute for Cancer Research. [a] Adjusted for age, study centre, education, parity, oral contraceptive use, hormone replacement therapy use (for postmenopausal women), smoking, non-alcoholic energy intake, family history of breast cancer, and diabetes. [b] Reference category.

Supplementary Table S3. Odds ratios (ORs) and corresponding 95% confidence intervals (CIs) for breast cancer according to the overall 2007 WCRF/AICR score. Italy and Switzerland, 1991-2008.

	**Cases (%)**	**Controls (%)**	**OR (95% CI)[a]**	**OR (95% CI)[b]**
Overall 2007 WCRF/AICR score[c]				
≤4	674 (23.2)	582 (18.1)	1.00[d]	1.00[d]
>4-<5	805 (27.7)	806 (25.0)	0.87 (0.74-1.01)	0.91 (0.78-1.06)
5-<5.75	846 (29.1)	947 (29.4)	0.78 (0.67-0.90)	0.82 (0.70-0.97)
≥5.75	582 (20.0)	884 (27.5)	0.60 (0.51-0.70)	0.67 (0.56-0.79)
p_{trend}			*<0.0001*	*<0.0001*
WCRF, a increment unit			0.82 (0.77-0.86)	0.85 (0.80-0.90)

WCRF/AICR, World Cancer Research Fund/American Institute for Cancer Research. [a] Adjusted for age, study centre, and education. [b] Adjusted for age, study centre, education, parity, menopausal status and age at menopause, oral contraceptive use, hormone replacement therapy use, smoking, non-alcoholic energy intake, family history of breast cancer, and diabetes. [c] The sum does not add up to the total because of missing values. [d] Reference category.
